# A novel ferroptosis-related gene signature associated with cell cycle for prognosis prediction in patients with clear cell renal cell carcinoma

**DOI:** 10.1186/s12885-021-09033-7

**Published:** 2022-01-03

**Authors:** Siteng Chen, Encheng Zhang, Tuanjie Guo, Jialiang Shao, Tao Wang, Ning Zhang, Xiang Wang, Junhua Zheng

**Affiliations:** 1 Department of Urology, Shanghai General Hospital, Shanghai Jiao Tong University School of Medicine, Shanghai, China; 2 Department of Urology, Ruijin Hospital, Shanghai Jiao Tong University School of Medicine, Shanghai, China

**Keywords:** Ferroptosis, Clear cell renal cell carcinoma, Prognosis, Cell cycle, Nomogram

## Abstract

**Background:**

It is of great urgency to explore useful prognostic markers for patients with clear cell renal cell carcinoma (ccRCC). Prognostic models based on ferroptosis-related gene (FRG) in ccRCC is poorly reported for now.

**Methods:**

Comprehensive analysis of 22 FRGs were performed in 629 ccRCC samples from two independent patient cohorts. We carried out least absolute shrinkage and selection operator analysis to screen out prognosis-related FRGs and constructed prognosis model for patients with ccRCC. Weighted gene co-expression network analysis was also carried out for potential functional enrichment analysis.

**Results:**

Based on the TCGA cohort, a total of 11 prognosis-associated FRGs were selected for the construction of the prognosis model. Significantly differential overall survival (hazard ratio = 3.61, 95% CI: 2.68–4.87, *p* < 0.0001) was observed between patients with high and low FRG score in the TCGA cohort, which was further verified in the CPTAC cohort with hazard ratio value of 5.13 (95% CI: 1.65–15.90, *p* = 0.019). Subgroup survival analysis revealed that our FRG score could significantly distinguish patients with high survival risk among different tumor stages and different tumor grades. Functional enrichment analysis illustrated that the process of cell cycle, including cell cycle-mitotic pathway, cytokinesis pathway and nuclear division pathway, might be involved in the regulation of ccRCC through ferroptosis.

**Conclusions:**

We developed and verified a FRG signature for the prognosis prediction of patients with ccRCC, which could act as a risk factor and help to update the tumor staging system when integrated with clinicopathological characteristics. Cell cycle-related pathways might be involved in the regulation of ccRCC through ferroptosis.

## Background

It is reported that in the United States, there will be 76,080 new renal malignancy cases and about 13,780 cases of death related to renal carcinoma in 2021 [1]. While in China, 74,000 new cases and a crude mortality of 1.97/10^5^ for renal cancer were estimated in 2015 [2]. Significant heterogeneity is found in different types of renal malignancy, among which, clear cell renal cell carcinoma (ccRCC) accounts for about 80% of malignant cases in renal [3]. As one of the most aggressive malignancies, ccRCC is responsible for most of the death cases caused by renal tumor [4]. Even for localized cases, about 25% of patients with ccRCC could also be troubled by tumor recurrence after receiving operative treatment [5]. Tumor staging system is currently the most fashionable method for survival prediction of patients with ccRCC. However, different survival outcomes could also be found in patients with similar tumor staging. Therefore, it is of great urgency to explore useful prognostic markers and develop novel prognostic models for patients with ccRCC.

Ferroptosis is a newfound process of programmed cell death, which differs from the traditional cell death processes since it is caused by the lethal accumulation of iron-dependent lipid hydroperoxides [6]. Current studies have also reported the important role of ferroptosis-related gene (FRG) in ccRCC. Through facilitating ferroptosis, SUV39H1 deficiency could restrain cell growth of ccRCC in vitro and in vivo [7]. Reduced expression of NCOA4, which is one of the FRG, was reported to be associated with tumor progression and poor prognosis of ccRCC [8]. In addition, cell density-regulated ferroptosis was found to be regulated via TAZ in cell death of renal cancer [9]. However, the prognostic model based on FRG in ccRCC is poorly reported for now.

Here, we preformed comprehensive analysis of FRG from two independent patient cohorts to develop and verify a prognostic model based on FRG and explored the potential mechanism underlying the FRG signature.

## Methods

### Patient cohorts and data sources

Two patient cohorts from The Cancer Genome Atlas (TCGA, https://portal.gdc.cancer.gov/) and Clinical Proteomic Tumor Analysis Consortium (CPTAC) [10] were recruited in this study. Only patients with complete gene expression data and clinical prognosis information were selected for analysis. The TCGA cohort included 531 ccRCC patients with pre-processed RNA-sequencing data and corresponding clinical data, which were retrieved from TCGA database. Ninety-eight patients in the CPTAC cohort with complete clinical data and processed RNA-seq sequencing data were also download from CPTAC database, including RNA-seq data of matched normal renal tissues. Basic clinicopathologic features were shown in Table 1. In addition, twenty-two ferroptosis-related genes were retrieved from the previous study [11].

**Table 1:** Basic clinical characteristics of patients in the TCGA cohort and CPTAC Cohort

	TCGA Cohort (531)	CPTAC Cohort (98)
**Age(years)**		
≥65	198(37.3%)	41(41.8%)
<65	333(62.7%)	57(58.2%)
**Sex**		
Male	345(65.0%)	75(76.5%)
Female	186(35.0%)	23(23.5%)
**Grade**		
G1	13(2.4%)	6(6.1%)
G2	229(43.1%)	47(48.0%)
G3	205(38.6%)	36(36.7%)
G4	76(14.3%)	9(9.2%)
Unknown	8(1.5%)	0
**Stage**		
I	266(50.1%)	46(46.9%)
II	57(10.7%)	11(11.2%)
III	124(23.4%)	31(31.6%)
IV	84(15.8%)	10(10.2%)
**T stage**		
T1	271(51.0%)	48(49.0%)
T2	69(13.0%)	12(12.2%)
T3	180(33.9%)	37(37.8%)
T4	11(2.1%)	1(1.0%)
**N stage**		
N1	16(3.0%)	/
N0	240(45.2%)	/
Unknown	275(51.8%)	/
**M stage**		
M1	79(14.9%)	/
M0	422(79.5%)	/
Unknown	30(5.6%)	/
**Survival**		
Dead	175(33.0%)	12(12.2%)
Living	356(67.0%)	86(87.8%)

### Development and verification of the prognosis model based on FRGs

Based on 22 FRGs in the TCGA cohort, we carried out least absolute shrinkage and selection operator (LASSO)-cox regression analysis by using *glmnet* package in R environment, which had been widely used in prognostic studies related to survival [12–15], to screen out survival-related FRGs. Their respective coefficients were also calculated corresponding to their weights in the prognosis model. We set the numbers of lambda as 1000 to ensure the robustness in the LASSO analysis. The FRG score was calculated as follow:

$$\mathrm{FRG}\ \mathrm{score}={\sum}_{i=1}^n\left(\mathrm{Coefi}\ast \mathrm{Feri}\right)$$

Coefi represents the coefficient of each prognosis-associated FRG, while Feri refers to the corresponding mRNA expression. We further verified the prognosis model in the independent CAPAC cohort, with cut-off value of the median value for each cohort.

### Constructing and evaluating a predictive nomogram combining FRG score and clinicopathologic factors

In order to construct a predictive nomogram for patients with ccRCC, we combined FRG score and clinicopathologic factors via *nomogramEx* and *rms* packages. Calibration with bootstrapping, receiver operating characteristic (ROC) curves with area under curve (AUC) value and decision curve were further carried out to evaluate the performance of the nomogram.

### Functional enrichment analysis

To explore the potential mechanism of the FRG score, we carried out weighted gene co-expression network analysis (WGCNA) to develop co-expression gene networks based on valid differentially expressed genes (DEGs, |fold change| ≥ 2, *p* < 0.01) in ccRCC, which were summarized in GEPIA2 [16]. Correlations between gene modules and predicted clinicopathologic features were calculated to find out the optimum module that was significantly associated with the FRG score. Finally, Kyoto Encyclopedia of Genes and Genomes (KEGG) pathway and Genetic Ontology (GO) analysis were carried out to explore the potential biological mechanisms in which the FRG signature might be involved via Metascape [17].

### Statistical analysis

In this study, R (3.6.2) were used for statistical analysis. Comparation of continuous variable between two groups was carried out through the Mann-Whitney U test. Comparation of continuous variable among more than two groups was carried out through the analysis of variance (ANOVA). Kaplan–Meier curve analysis was performed by using log-rank test to compare overall survival (OS). Pearson correlation analysis was also performed to calculate the correlation coefficient between two variables.

## Results

### Developed and verified the prognosis model based on FRGs

**Fig. 1:**
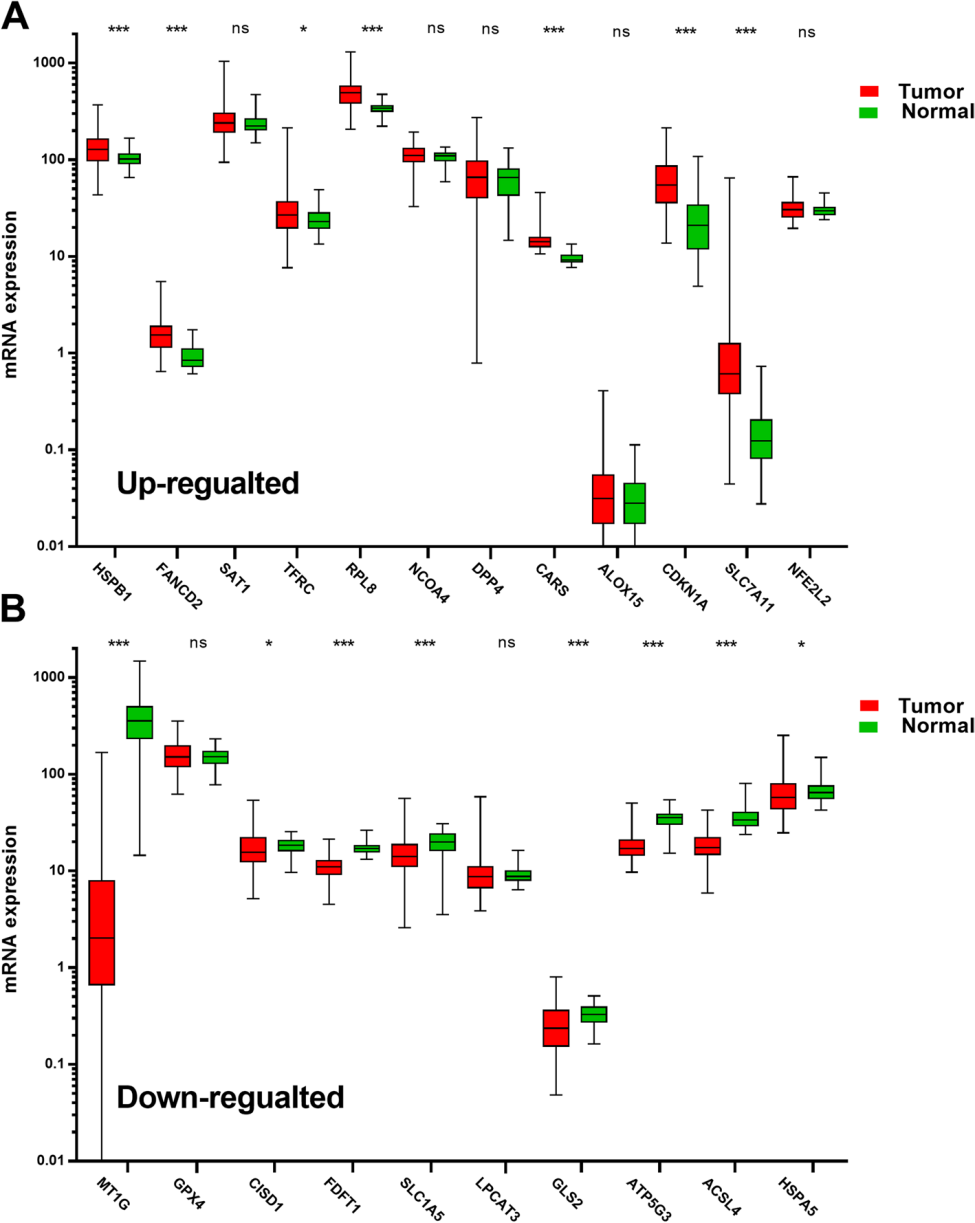
Differential expressions of ferroptosis-related genes between clear cell renal cell carcinoma and normal renal tissue. **A** The up-regulated ferroptosis-related genes in clear cell renal cell carcinoma compared with normal renal tissue. **B** The down-regulated ferroptosis-related genes in clear cell renal cell carcinoma compared with normal renal tissue

We firstly identify the important roles of FRGs in ccRCC from the CAPAC cohort. As shown in Fig. 1A, 7 FRGs (HSPB1, FANCD2, TFRC, RPL8, CARS, CDKN1A and SLC7A11) were significantly up-regulated in tumor samples, while 8 FRGs, including MT1G, CISD1, FDFT1, SLC1A5, GLS2, ATP5G3, ACSL4 and HSPA5, were found to be significantly down-regulated in ccRCC (Fig. 1B).

**Fig. 2:**
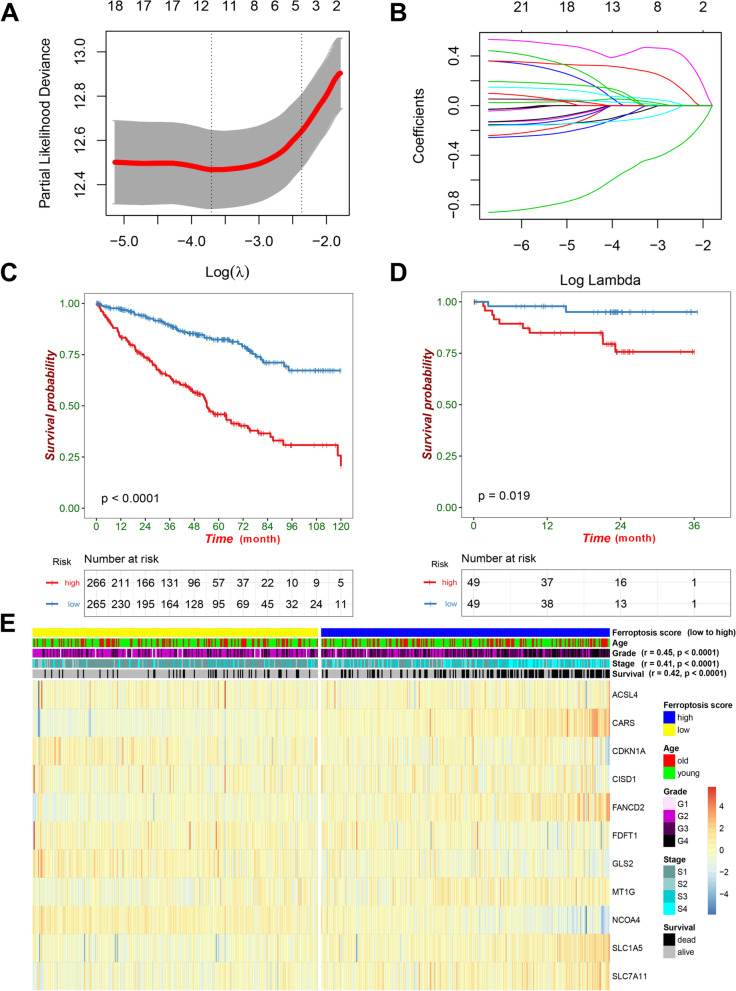
Prognosis model based on ferroptosis-related genes for ccRCC. **A**, **B** The tenfold cross-validated error and coefficients at varying levels of penalization plotted against the log (lambda) sequence for the least absolute shrinkage and selection operator analysis, respectively. **C** Kaplan-Meier survival analysis of overall survival stratified by FRG score for ccRCC patients in the TCGA cohort. **D** Kaplan-Meier survival analysis of overall survival stratified by FRG score in another validation CPTAC cohort. **E** Heatmap illustrated the expression of the selected genes and the distribution of clinicopathologic factors in the TCGA cohort. ccRCC, clear cell renal cell carcinoma; FRG, ferroptosis-related gene; TCGA, the cancer genome atlas; CPTAC, clinical proteomic tumor analysis consortium; r, Pearson correlation coefficient; G1, grade 1; G2, grade 2; G3, grade 3; G4, grade 4; S1, stage i; S2, stage ii; S3, stage iii; S4, stage iv

Based on the TCGA cohort, LASSO-cox regression analysis screened out 11 prognosis-associated FRGs for the construction of the prognosis model, including CARS, FANCD2, ACSL4, CISD1, SLC1A5, SLC7A11, MT1G, CDKN1A, FDFT1, GLS2 and NCOA4 (Fig. 2a, b). The selected genes and their respective coefficients were shown in Table 2. The FRG score was calculated as mentioned in the method part. Significantly differential OS (hazard ratio = 3.61, 95% CI: 2.68–4.87, *p* < 0.0001) was observed between patients with high and low FRG score in the TCGA cohort (Fig. 2c), which was further verified in the CPTAC cohort with hazard ratio value of 5.13 (95% CI: 1.65–15.90, *p* = 0.019, Fig. 2d). As shown in Fig. 2e, the heatmap and correlation analysis indicated that higher FRG score was significantly associated with higher rate of death (r = 0.42, *p* < 0.0001), higher tumor stage (r = 0.41, p < 0.0001), and higher tumor stage (r = 0.45, p < 0.0001).

**Table 2:** Selected features and associated weights in the prognosis model from the TCGA cohort

Genes	Weights
CARS	0.415747
FANCD2	0.303476
ACSL4	0.095466
CISD1	0.068751
SLC1A5	0.068244
SLC7A11	0.048827
MT1G	0.024091
CDKN1A	−0.07425
FDFT1	−0.0774
GLS2	−0.11893
NCOA4	−0.53171

### The FRG score could act as a prognostic factor for patients with ccRCC

**Fig. 3:**
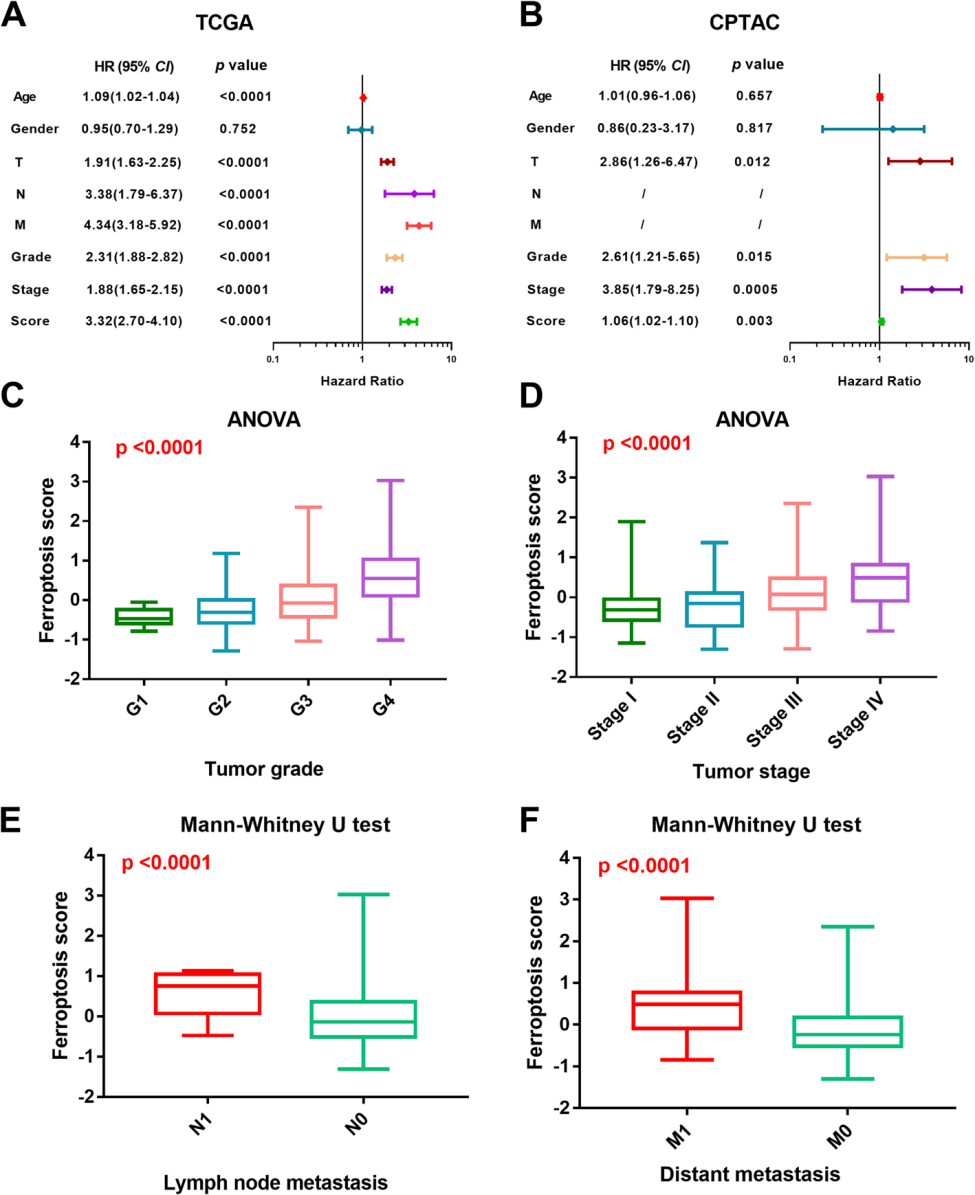
Evaluation of the ferroptosis-related prognosis model. **A**-**B** Univariate cox regression analyses of FRG score and clinicopathologic factors in the TCGA cohort and CPTAC cohort, respectively. **C**-**F** The different distributions of FRG score among different tumor grades, tumor stages, lymph node metastasis status and distant metastasis status. TCGA, the cancer genome atlas; FRG, ferroptosis-related gene; CPTAC, clinical proteomic tumor analysis consortium; ANOVA, analysis of variance

We next to perform univariate cox regression analysis to further evaluate the prediction performance of the FRG score. As shown in Fig. 3a, the FRG score could act as a risk factor for survival prediction of ccRCC patient in the TCGA cohort, which was further verified in the CPTAC cohort (Fig. 3b). Further comparative analyses revealed that the patients with high tumor grade (*P* < 0.0001, Fig. 3c) or high tumor stage (P < 0.0001, Fig. 3d) tended to have higher level of FRG score. In addition, higher level of FRG score was also found in patients with tumor lymph node metastasis status (P < 0.0001, Fig. 3e) or tumor distant metastasis status (P < 0.0001, Fig. 3f).

**Fig. 4:**
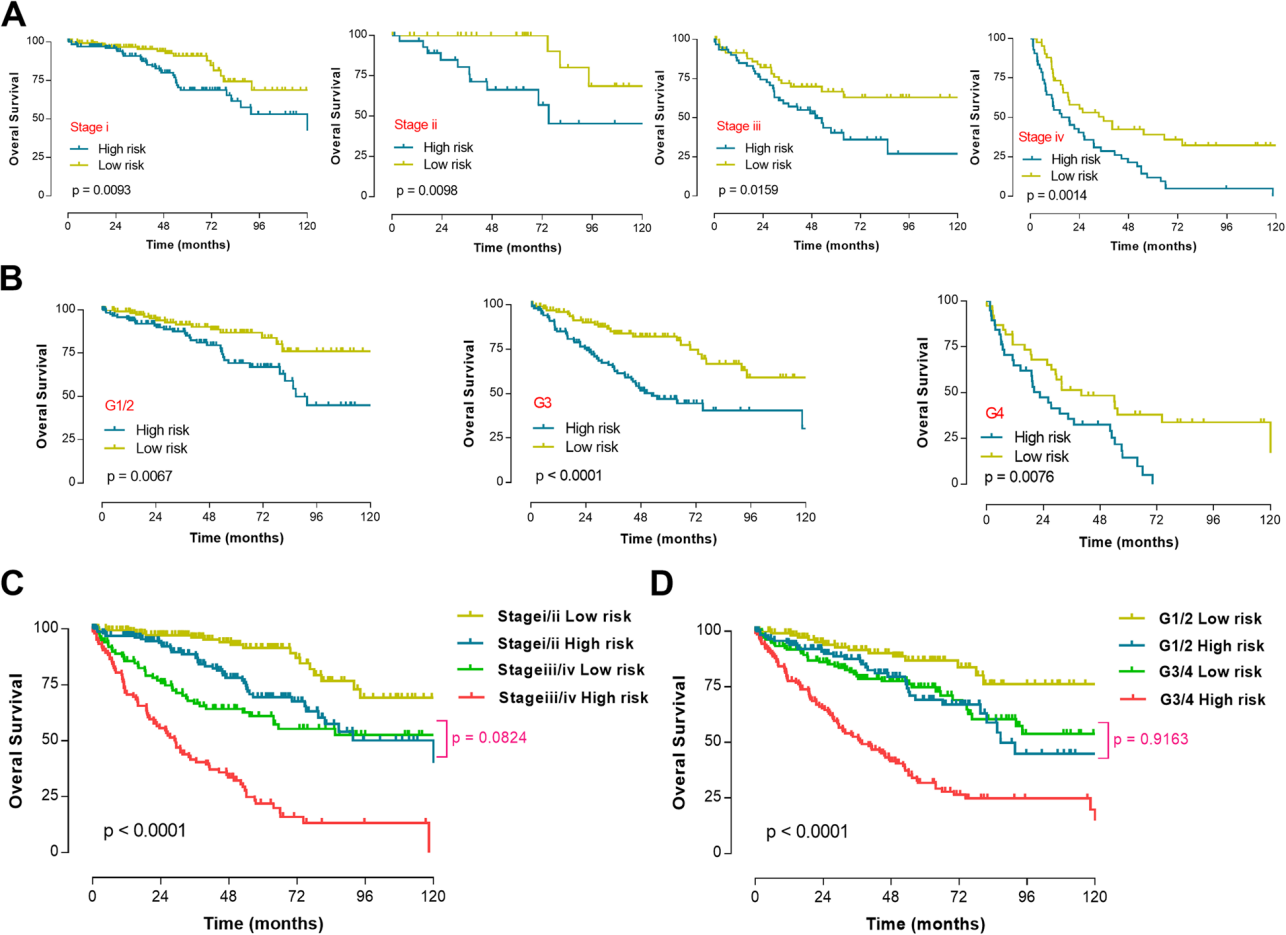
Subgroup survival analysis of the ferroptosis-related prognosis model in the TCGA cohort. **A** Subgroup survival analysis among different tumor stages. **B** Subgroup survival analysis among different tumor grades. **C** New tumor staging system based on current staging system and the ferroptosis-related prognosis model. **D** New tumor grading system based on current grading system and the ferroptosis-related prognosis model. TCGA, the cancer genome atlas

Subgroup survival analysis of the prognosis model in the TCGA cohort revealed that our FRG score could significantly distinguish patients with high survival risk among different tumor stages (Fig. 4a) and different tumor grades (Fig. 4b). New tumor staging system based on current staging system and the FRG score performed well in distinguishing ccRCC patients with different clinical prognoses (Fig. 4c). Patients with stage i-ii/high risk score tumors had similar survival outcomes compared to patients with stage iii-iv/low risk score tumors (*p* = 0.0824). In addition, patients with grade 1–2/high risk score tumors also had similar survival outcomes when compared to patients with grade 3–4/low risk score tumors (*p* = 0.9163, Fig. 4d).

### Improved prognostic accuracy of the FRG score integrated with clinicopathologic features

**Fig. 5:**
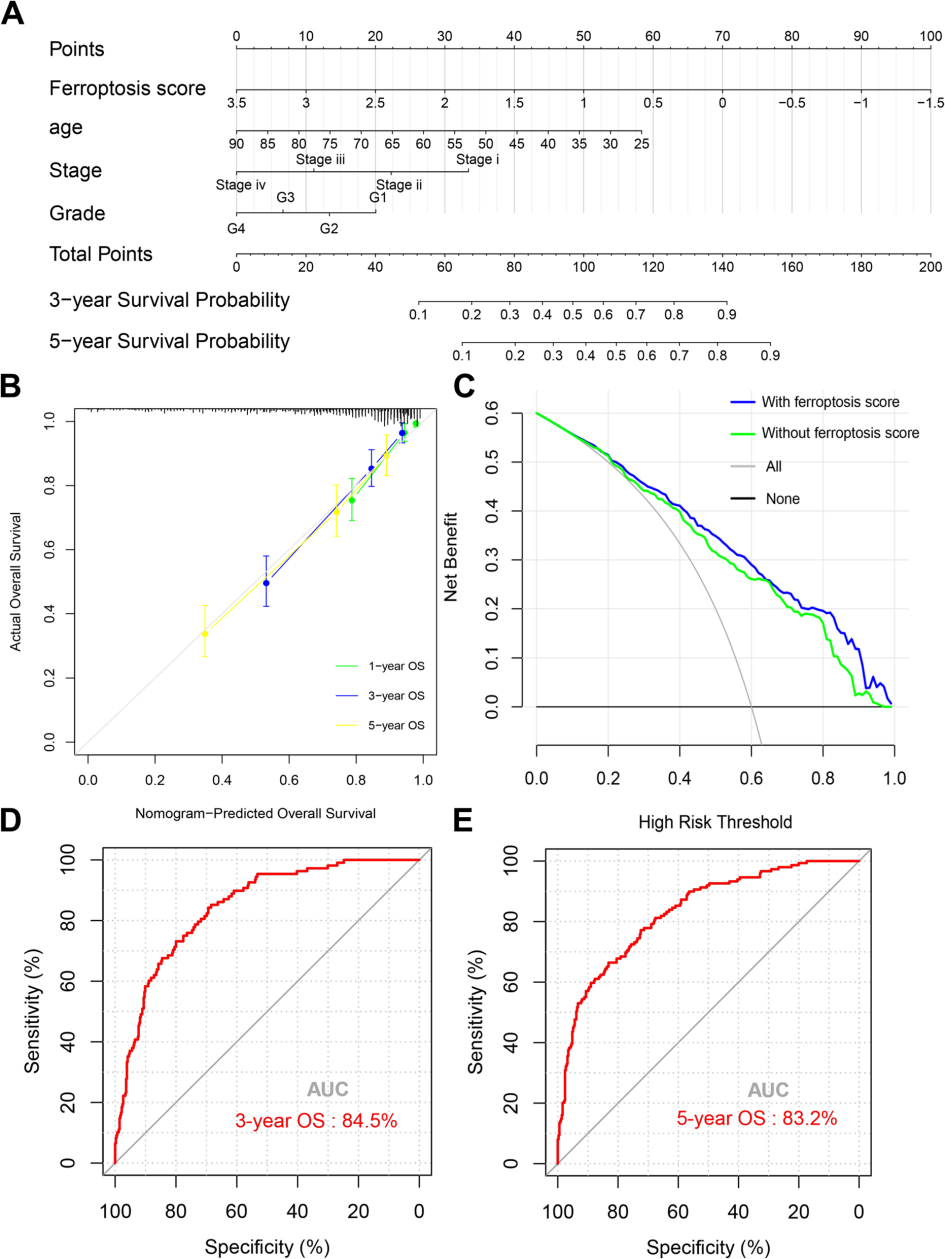
Construction and evaluation of a predictive nomogram in the TCGA cohort. **A** Nomogram based on FRG score and clinicopathologic factors for OS prediction of ccRCC patients. **B** Evaluation of the prognostic nomogram model for 1-, 3- and 5-year OS prediction. **C** Decision curve analysis compared OS benefits among the nomogram with or without FRG score. **D**, **E** ROC curve of 3-, and 5-year OS prediction based on the prognostic nomogram, respectively. TCGA, the cancer genome atlas; OS, overall survival; FRG, ferroptosis-related gene; ccRCC, clear cell renal cell carcinoma; ROC, receiver operating characteristic; AUC, area under curve

To explore whether the accuracy of the prognosis model could be improved through combining our FRG score and clinicopathologic features, we developed an integrated nomogram based on the FRG score, patient age, tumor grade and tumor stage (Fig. 5a). The calibration analysis indicated that the survival rate predicted by the nomogram had excellent agreement with actual observations at 1-, 3- and 5-year follow up (Fig. 5b). Further decision curve analysis verified the improved prognostic accuracy via the FRG score (Fig. 5c). ROC curve analyses illustrated that that AUC of the nomogram for survival prediction in 3- and 5-year reached to 84.5 and 83.2%, respectively (Fig. 5d, e).

### Cell cycle-related pathways were associated with the FRG score in ccRCC

**Fig. 6:**
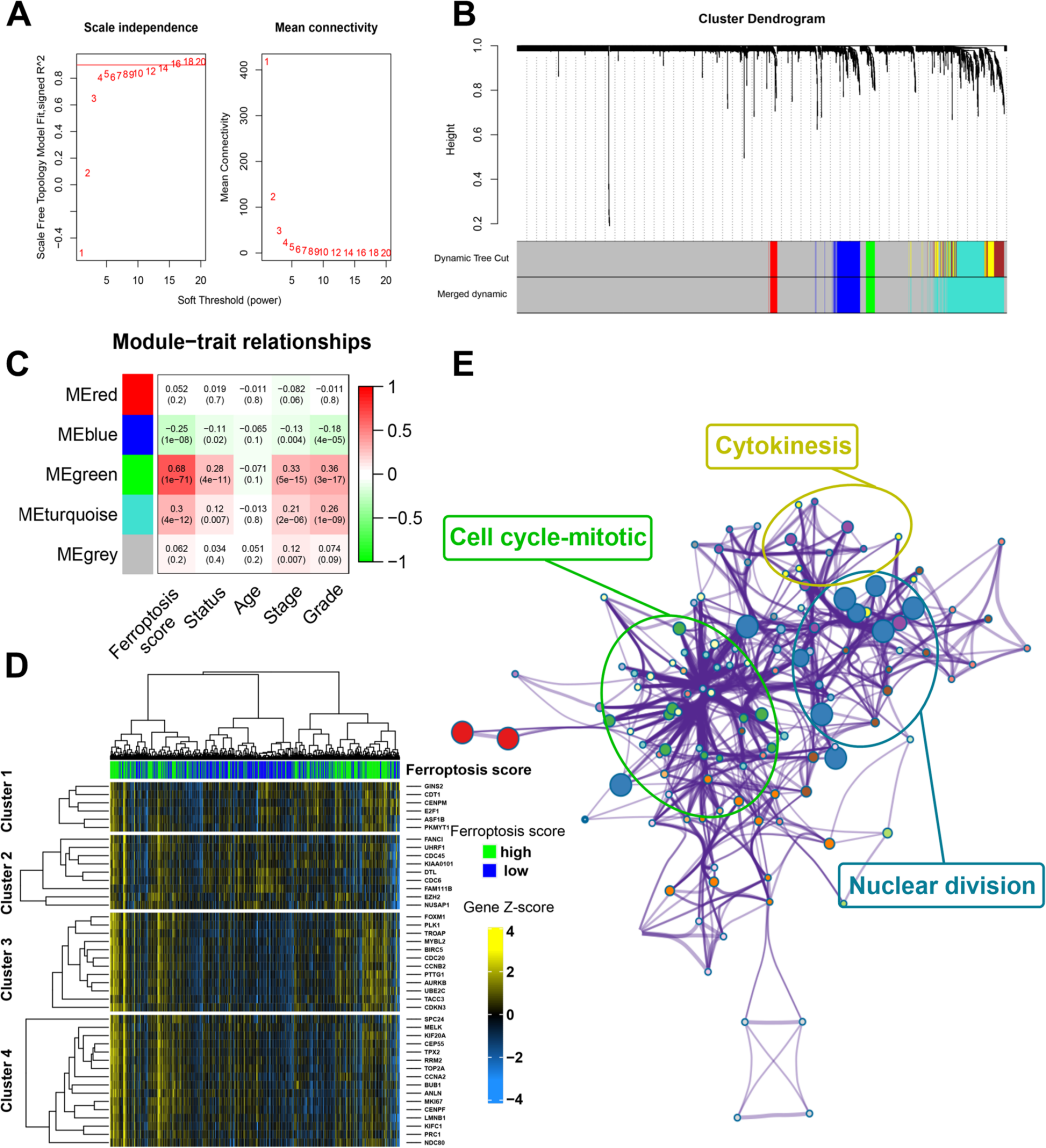
WGCNA and potential mechanism analysis from co-expressed genes associated with the FRG score. **A** Soft power estimation in ccRCC for WGCNA. **B** Gene dendrogram with different colors showing the modules identified by WGCNA. **C** The relationship between gene modules and clinical characteristic. **D** Heatmap visualizing the expressions of the co-expressed genes in green module. **E** Potentially enriched pathways of the co-expressed genes in green module. WGCNA, weighted gene co-expression network analysis; ccRCC, clear cell renal cell carcinoma

A total of 2353 DEGs were analyzed through WGCNA in this study. According to the recommendation of *pickSoftThreshold*, the soft-thresholding power of *β* value was set as 18 (Fig. 6a). All the DEGs associated with ccRCC were then hierarchically clustered into 5 gene modules (Fig. 6b). As shown in Fig. 6c, correlation analysis indicated that the green model (MEgreen) seemed to have the highest correlation with FRG score. The heatmap also illustrated the relationships of the 43 classified genes of the green model and the FRG score (Fig. 6d). Further functional enrichment analysis revealed that our FRG score might involve in pathways associated with the process of cell cycle, including cell cycle-mitotic pathway, cytokinesis pathway and nuclear division pathway (Fig. 6e).

## Discussion

Due to the significant heterogeneity and aggressiveness of ccRCC, different clinical outcomes could still be found in patients with similar tumor stage or grade. Therefore, it is of great urgency to find out novel prognostic markers for clinical practices. Fortunately, high-throughput genetic techniques for oncology have revolutionized the development of prognostic biomarkers for malignant tumor [10, 18].

Ferroptosis is closely related to tumor invasion and metastasis. It was reported that tumor-infiltrating CD8^+^ T cells with CD36 deficiency had low expression of FRGs, and CD36 deficiency had been confirmed to be associated with reduced ferroptosis in tumor-infiltrating CD8^+^ T cells [19]. A recent study has also revealed that oleic acid could protect melanoma cells from ferroptosis through acsl3-dependent manner. In addition, melanoma cells from lymph nodes were more resistant to ferroptosis [20].

Here, we developed and verified a prognostic model based on FRGs from two independent patient cohorts. Cox regression analysis revealed that the FRG score could act as a survival risk factor for patients with ccRCC. Improved prognostic accuracy was also found in the nomogram integrated with FRG score and clinicopathologic features, which had excellent agreements with the actual survival rates at 1-, 3- and 5-year follow up.

The new tumor staging system based on current staging system and the ferroptosis-related prognosis model was found to act better in distinguishing ccRCC patients with worse prognosis. Patients with low tumor stage (stage i-ii) might be faced with similar survival risk to patients with high tumor stage (stage iii-iv) if they were accompanied with high FRG score, which might account for the clinical observations that different survival outcomes could sometime be found in patients with similar tumor staging, indicating the potential application value of our ferroptosis-based prognosis model in clinical practices.

A total of 43 core genes clustered in the green model were found to be significantly associated with our FRG score. Further functional enrichment analysis revealed that our FRG score might involve in pathways associated with the process of cell cycle in ccRCC. It was reported that ferroptosis could be regulated by p53, which was an indispensable regulator of the cell cycle and could enhance ferroptosis by inhibiting SLC7A11. In addition, p53 could also inhibit ferroptosis by directly inhibiting the activity of DPP4 or inducing the expression of CDKN1A/p21 [21].

Several limitations could still be found in this study. Firstly, cross-validations among two independent patient cohorts were carried out in this study, however, potential bias might still exist since retrospective public cohorts were used for analyses. Secondly, prospective single- or multi-center studies are still wanted for further verifying the ferroptosis-related prognostic model. Finally, even though our study revealed that cell cycle-related pathways were associated with the FRG score in ccRCC, experimental studies for potential mechanism exploring and function verification are still needed for subsequent analyses.

## Conclusions

We developed and verified a FRG signature for the prognosis prediction of patients with ccRCC, which could act as a risk factor and help to update the tumor staging system when integrated with clinicopathological characteristics. Cell cycle-related pathways might be involved in the regulation of ccRCC through ferroptosis, which still need further experimental studies for function verifications of the study.

## Data Availability

The raw data used in this study could be downloaded from the TCGA (https://portal.gdc.cancer.gov/) and the CPTAC (https://cptac-data-portal.georgetown.edu/) databases.

## Abbreviations

ccRCC: Clear-cell renal cell carcinoma
FRG: Ferroptosis-related gene
TCGA: The Cancer Genome Atlas
CPTAC: Clinical Proteomic Tumor Analysis Consortium
LASSO: Least absolute shrinkage and selection operator
ROC: Receiver operating characteristic
AUC: Area under curve
WGCNA: Weighted gene co-expression network analysis
DEGs: Differentially expressed genes
KEGG: Kyoto Encyclopedia of Genes and Genomes
GO: Genetic Ontology
OS: Overall survival
